# Supplementary material for: Membrane marker selection for segmenting single cell spatial proteomics data
Source: Nat Commun. 2022 Apr 14;13:1999. doi: 10.1038/s41467-022-29667-w (PMC9010440; doi:10.1038/s41467-022-29667-w)
Supplement: Supplementary file 1 — Supplementary Information [file 41467_2022_29667_MOESM1_ESM.pdf]

Membrane marker selection for segmenting single cell spatial proteomics data: Supplementary Information

Monica T. Dayao[1,2], Maigan Brusko[3], Clive Wasserfall[3], and Ziv Bar-Joseph[2,4,*]

[1]Joint Carnegie Mellon University-University of Pittsburgh Ph.D. Program in Computational Biology

[2]Computational Biology Department, School of Computer Science, Carnegie Mellon University

[3]Department of Pathology, Immunology and Laboratory Medicine, University of Florida

[4]Machine Learning Department, School of Computer Science, Carnegie Mellon University

[*]Correspondence: Ziv Bar-Joseph, zivbj@cs.cmu.edu

Dataset	HuBMAP ID	Tissue	No. Tiles	No. Z-planes	No. Distinct Proteins	Training/ Cross-val	Eval
1	N/A (from [1])	murine spleen	63	15	31	✓	
2	HBM869.VZJM.366	lymph node	25	20	19	✓	
3	HBM432.LLCF.677	spleen	63	12	19	✓	
4	HBM588.FHDS.363	thymus	81	13	19	✓	
5	HBM279.TQRS.775	lymph node	25	10	19		✓
6	HBM337.FSXL.564	spleen	63	17	19		✓
7	HBM376.QCCJ.269	thymus	117	12	19		✓
8	HBM754.WKLP.262	lymph node	63	12	29		✓
9	HBM556.KSFB.592	spleen	63	17	29		✓
10	HBM288.XSQZ.633	thymus	63	12	29		✓
11	N/A [2]	bone marrow	1	N/A	55		✓

Supplementary Table 1: Datasets used for training and evaluation of the RAMCES CNN model. 'Training/Cross-val' refers to datasets used to train the model for cross-validation. 'Eval' refers to datasets used for the rest of the results. Datasets from HuBMAP can be found from the HuBMAP data portal, along with the respective donor demographic information. The source data for dataset 11 from [2] was from the 'Multi-tumor TMA' data, region 4. This data was already pre-processed with its optimal z-plane selected.

	Datasets				
	1	2-7	8-10	11	
1	DAPI	DAPI	DAPI	DRAQ5	Pan-Cytokeratin
2	CD45	CD31	CD31	CD79a	CD68
3	Ly6C	CD8	CD8	FOXP3	CD3
4	TCR	CD45	CD20	CDX2	SMA
5	Ly6G	CD20	Ki67	CD8	CD34
6	CD19	Ki67	CD3e	p53	EpCAM
7	CD169	CD3e	SMActin	GATA3	CollagenIV
8	CD106	Actin	Podoplanin	CD21	CD45RO
9	CD3	Podoplanin	CD68	PD-L1	Podoplanin
10	CD16/32	CD68	PAN-CK	Ki67	CD15
11	CD8a	PAN-CK	CD21	CD45	CD7
12	CD90	CD21	CD4	CD30	CD163
13	F4/80	CD4	Lyve1	PAX5	ChromograninA
14	CD11c	CD45R0	CD45R0	HLA-DR	CD31
15	TER-119	CD11c	CD11c	CD5	CD123
16	CD11b	ECAD	CD35	CD2	MMP9
17	IgD	CD107a	ECAD	CD45RA	CD138
18	CD27	CD44	CD107a	CD4	CD38
19	CD5	Histone H3	CD34	PD-1	Hyaluronan
20	CD79b		CD44	MUC-1	
21	CD71		HLA-DR	BCL2	
22	CD31		FoxP3	CD56	
23	CD4		CD163	Cytokeratin7	
24	IgM		Collagen IV	CD25	
25	B220		Vimentin	VISTA	
26	ER-TR7		CD15	Hep-Par-1	
27	HMCII		CD45	CD11c	
28	CD35		CD5	IRF4	
29	CD21/35		CD1c	CD20	
30	CD44			EGFR	
31	NKp46			IDO-1	
32				GranzymeB	
33				MelanA	
34				OX-40	
35				Vimentin	
36				Synaptophysin	
37				CD117	
38				Na-K-ATPase	
39				CD194	
40				CD57	

Supplementary Table 2: List of markers profiled in each CODEX dataset. The dataset number refers to the number in Supplementary Table 1. For antibody information for datasets 2-10, see Supplementary Table 10.

Dataset 1		Datasets 2-4	2	3	4
Protein/Marker	Label	Protein/Marker	Labels		
DAPI	0	DAPI	0	0	0
CD45	1	CD31	0	0	0
Ly6C	0	CD8	0	0	1
TCR	1	CD45	1	1	0
Ly6G	0	CD20	0	1	0
CD19	0	Ki67	0	0	0
CD169	0	CD3e	1	1	1
CD106	0	Actin	0	0	0
CD3	1	Podoplanin	0	0	0
CD16/32	0	CD68	0	0	0
CD8a	1	PAN-CK	0	0	0
CD90	1	CD21	0	1	0
F4/80	0	CD4	1	1	1
CD11c	1	CD45R0	0	1	0
TER-119	0	CD11c	0	0	0
CD11b	0	ECAD	0	1	0
IgD	1	CD107a	0	0	0
CD27	0	CD44	0	1	0
CD5	1	Histone H3	0	0	0
CD79b	1				
CD71	0				
CD31	1				
CD4	1				
IgM	0				
B220	0				
ER-TR7	0				
HMCII	0				
CD35	0				
CD21/35	0				
CD44	0				
NKp46	0				

Supplementary Table 3: Labeling of each marker in the training datasets. 1 indicates a that the marker labeled membranes well in the specified dataset, and 0 indicates that the marker labeled other cellular/extra-cellular components.

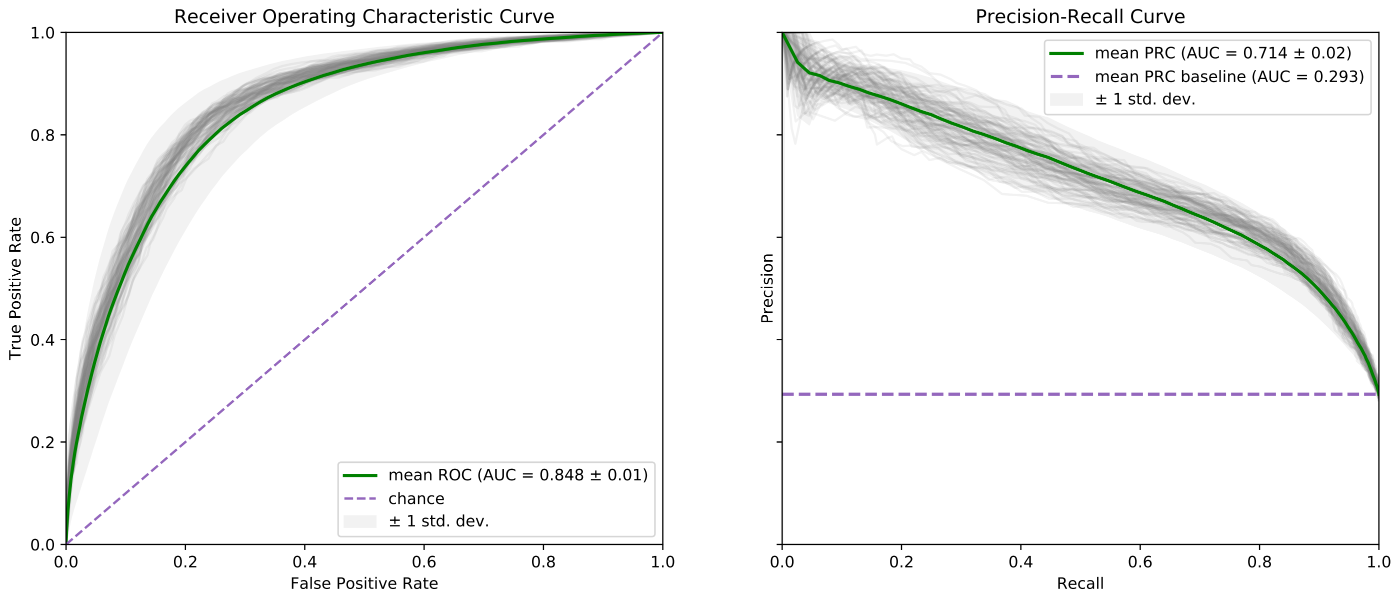

Supplementary Figure 1: ROC and PR curves for CNN models trained on 100 different bootstrap samples from datasets 1-4 (Supplementary Table 1). The gray lines show the curves for each individual bootstrap model. The mean ROC and PR curves (green) are presented with error bands representing ± standard deviation ($n = 100$ bootstrap models). The purple dashed lines represent the ROC and PRC baselines.

	Lymph node						Spleen					
Rank	Dataset 5			8			6			9		
1	**CD4***	0.747	0.817	**CD8***	0.964	0.223	**CD45***	0.995	0.048	**CD20***	0.787	0.747
2	**CD45***	0.711	0.868	**CD4***	0.963	0.227	**CD45R0***	0.994	0.053	Vimentin	0.625	0.955
3	**CD20***	0.683	0.901	**CD3e***	0.963	0.228	**CD3e***	0.993	0.060	SMActin	0.376	0.956
4	**CD3e**	0.668	0.917	**CD20**	0.958	0.250	**CD4**	0.990	0.082	**CD107a**	0.252	0.815
5	**CD45R0**	0.661	0.924	**CD45**	0.899	0.471	**CD20**	0.989	0.088	**CD163**	0.228	0.775
	Thymus						Bone marrow					
Rank	7			10			11					
1	**CD3e***	0.993	0.060	**CD3e***	0.935	0.347	**CD8***	0.934	0.352			
2	**CD8***	0.963	0.230	**CD8***	0.924	0.386	**CD34***	0.793	0.735			
3	**CD4***	0.945	0.308	**CD45***	0.899	0.471	**HLA-DR***	0.736	0.832			
4	**CD20**	0.862	0.579	**CD4**	0.886	0.512	CD57	0.718	0.859			
5	**ECAD**	0.697	0.884	**CD1c**	0.872	0.551	**CD45RA**	0.699	0.883			

Supplementary Table 4: Top 5 ranked proteins for datasets 5-11 using the CNN model trained on datasets 2-4 (Supplementary Table 1). Bolded protein name means that it is labeled as a membrane protein by the Human Protein Atlas [3]. An asterisk (*) means that it was selected to use for segmentation. For each protein, there are two numbers in the same row. The first number is the score output by RAMCES for that protein. The second number is the Shannon entropy, which can be interpreted as the uncertainty of the RAMCES model (Methods).

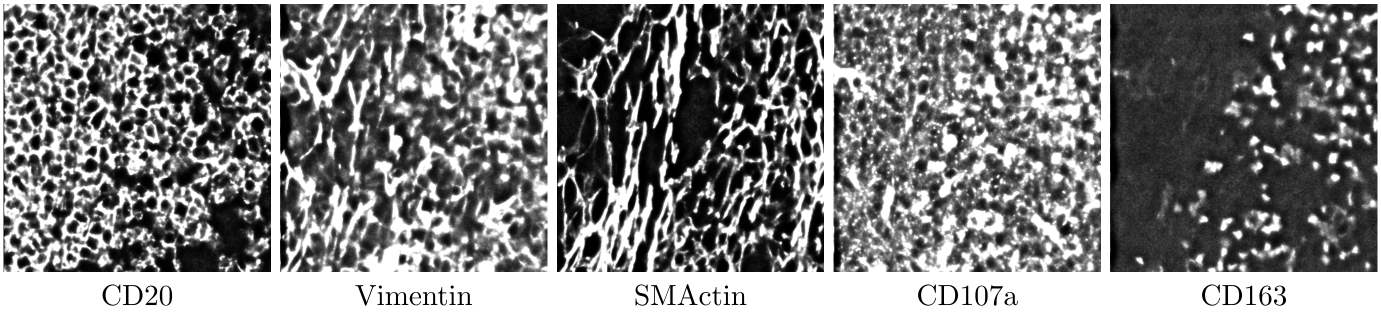

Supplementary Figure 2: An example field of view for the top 5 ranked proteins for spleen dataset 9. Visually, only CD20 labels cell membranes well.

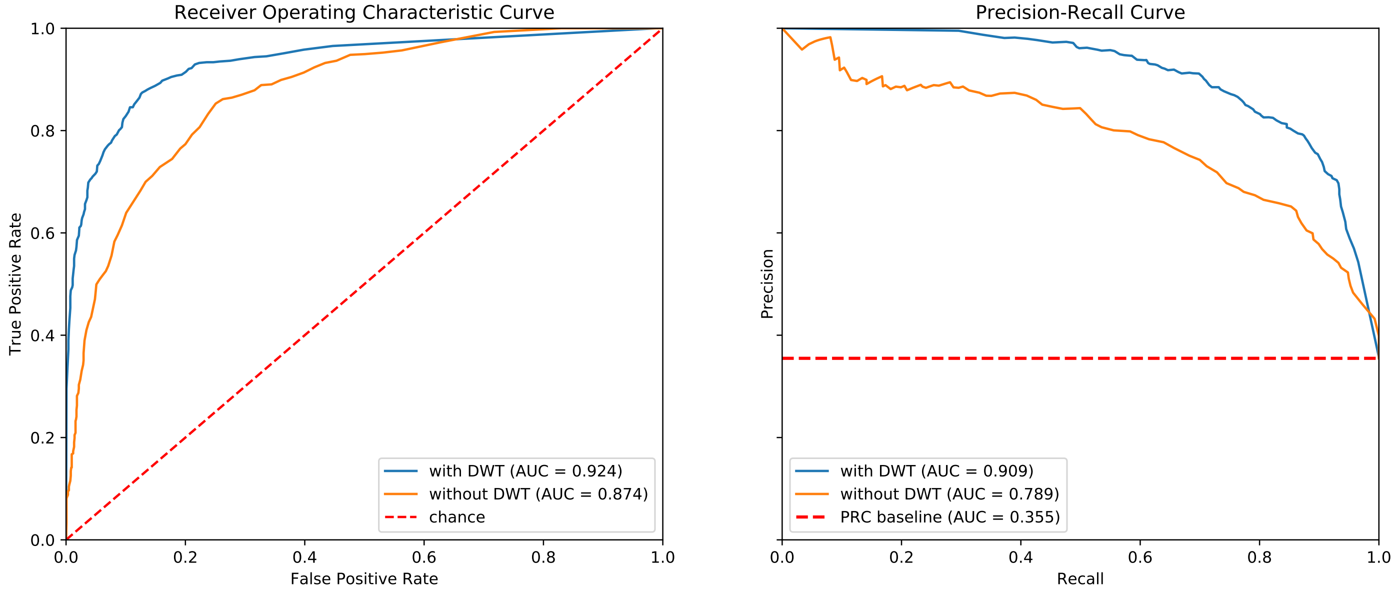

Supplementary Figure 3: Reciever operating characteristic (ROC) and precision-recall (PR) curves comparing CNN performance with and without using the DWT. This evaluation was performed on the Goltsev et al. dataset [1].

Tissue (dataset ID)	Total no. cells	CD3+,CD4+		CD3+,CD8+		CD4+,CD8+		CD68+,CD4-,CD8-	
		Nucl-ext	Top 3	Nucl-ext	Top 3	Nucl-ext	Top 3	Nucl-ext	Top 3
Lymph node (5)	46840	15.2%	21.9%	7.3%	9.0%	3.2%	4.2%	1.5%	3.3%
Spleen (6)	123202	1.7%	2.8%	0.8%	1.3%	0.4%	0.9%	0.4%	0.7%
Thymus (7)	264898	-	-	-	-	1.5%	1.6%	-	-

Supplementary Table 5: Percentages of cells coexpressing proteins using different segmentations. 'Nucl-ext' refers to the default Cytokit [4] nucleus extension method, and 'Top 3' refers to our method with the top 3 proteins combined as the membrane marker.

Tissue (dataset ID)	Lymph Node (5)		Spleen (6)		Thymus (7)	
Comparison	Jaccard	Dice	Jaccard	Dice	Jaccard	Dice
Top3 (combined) vs rank1	0.8809	0.9362	0.9081	0.9511	0.8456	0.9144
Top3 (combined) vs rank2	0.9167	0.9564	0.9651	0.9821	0.9444	0.9711
Top3 (combined) vs rank3	0.8213	0.9011	0.9105	0.9529	0.8636	0.9252
Avg top3 vs rank#	0.8730	0.9312	0.9279	0.9620	0.8845	0.9369
Rank1 vs rank2	0.8434	0.9148	0.8878	0.9396	0.8203	0.8989
Rank1 vs rank3	0.7458	0.8539	0.8800	0.9356	0.8521	0.9179
Rank2 vs rank3	0.8237	0.9026	0.8871	0.9396	0.8265	0.9025
Avg rank# vs rank #	0.8043	0.8904	0.8850	0.9383	0.8329	0.9064

Supplementary Table 6: Cell segmentation mask overlap (calculated by the Jaccard index and Dice coefficient) between different segmentation methods for datasets 5-7 (Supplementary Table 1). Top3 corresponds to the RAMCES segmentation using the top 3 combined membrane markers. Rank1, rank2 and rank3 correspond to the segmentation using the first, second and third-ranked individual (not combined) protein channels, respectively. The 'Avg top3 vs rank#' row is the average of the Top3 segmentation overlapping with the individual channel segmentations. The 'Avg rank# vs rank #' row shows the average pairwise overlaps between the individual channel segmentations.

	Jaccard Index			Dice Coefficient		
Comparison w/ manual segmentation	Tile 1	Tile 2	Avg	Tile 1	Tile 2	Avg
Top2 (combined)	0.6035	0.6985	0.6510	0.7527	0.8225	0.7876
Top3 (combined)	0.6043	0.6930	0.6487	0.7533	0.8187	0.7860
Top4 (combined)	0.6037	0.6972	0.6505	0.7529	0.8216	0.7873
Rank1	0.5917	0.6592	0.6255	0.7434	0.7946	0.7690
Rank2	0.6152	0.7095	0.6624	0.7618	0.8301	0.7951
Rank3	0.5607	0.6743	0.6175	0.7185	0.8054	0.7611
Avg rank#	0.5892	0.6810	0.6351	0.7412	0.8100	0.7756
Nucl-ext	0.5434	0.6569	0.6002	0.7042	0.7922	0.7482

Supplementary Table 7: Cell segmentation mask overlap (calculated by the Jaccard index and Dice coefficient) with the first expert manual annotation of two image tiles from dataset 5 (Supplementary Table 1). Top2, top3 and top4 correspond to the RAMCES segmentation using the top 2,3,4 combined membrane markers, respectively. The Top3 segmentation was used for the analysis in the main text. Rank1, rank2 and rank3 correspond to the segmentation using the first, second and third-ranked individual (not combined) protein channels. The 'Avg rank#' row is the average value for those individual protein segmentations. The 'Nucl-ext' row corresponds to the default nucleus extension segmentation method from Cytokit.

	Jaccard Index			Dice Coefficient		
Comparison w/ manual segmentation	Tile 1	Tile 2	Avg	Tile 1	Tile 2	Avg
Top2 (combined)	0.5110	0.4752	0.4931	0.6764	0.6442	0.6603
Top3 (combined)	0.5110	0.4926	0.5018	0.6764	0.6600	0.6682
Top4 (combined)	0.5099	0.4837	0.4968	0.6752	0.6520	0.6636
Rank1	0.5019	0.4711	0.4865	0.6683	0.6405	0.6544
Rank2	0.5184	0.4926	0.5055	0.6828	0.6600	0.6714
Rank3	0.4932	0.4661	0.4797	0.6606	0.6358	0.6482
Avg rank#	0.5045	0.4766	0.4906	0.6706	0.6454	0.6580
Nucl-ext	0.4347	0.3956	0.4152	0.6060	0.5669	0.5865

Supplementary Table 8: Cell segmentation mask overlap with the second expert manual annotation of two image tiles from dataset 5 (Supplementary Table 1). Format is the same as in Supplementary Table 7.

	Jaccard Index			Dice Coefficient		
	Tile 1	Tile 2	Avg	Tile 1	Tile 2	Avg
Agreement between manual segmentations	0.5570	0.5174	0.5372	0.7155	0.6819	0.6987

Supplementary Table 9: Cell segmentation mask overlap between the two expert annotations of two tiles in dataset 5 (Supplementary Table 1), measured by the Jaccard Index and Dice Coefficient.

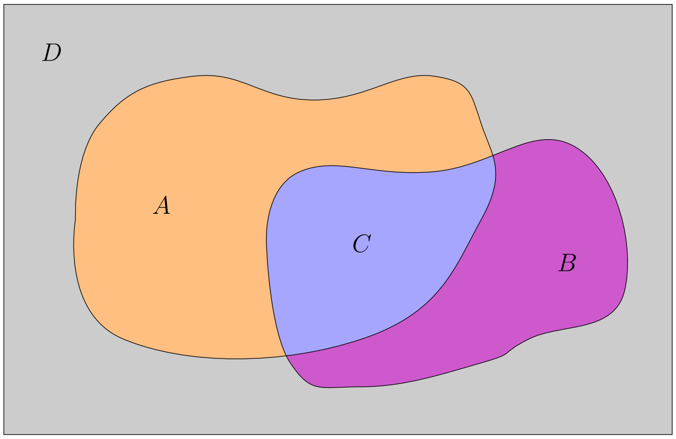

Supplementary Figure 4: Visualization of agreement and disagreement areas for two different segmentation methods. Let $A \cup C$ be the segmentation area for method #1. Let $B \cup C$ be the segmentation area for method #2. C is referred to as the agreement in the foreground between methods #1 and #2. D is referred to as the agreement in the background. A is the area where method #1 assigns to inside of cells and method #2 assigns to background, and vice versa for B. If method #1 is the more accurate segmentation method, we would expect that the average biomarker distribution for area A would be more similar to the distribution for area C than the distribution for area B is to C's distribution. We would also expect that B's distribution is more similar to the distribution for area D, the background.

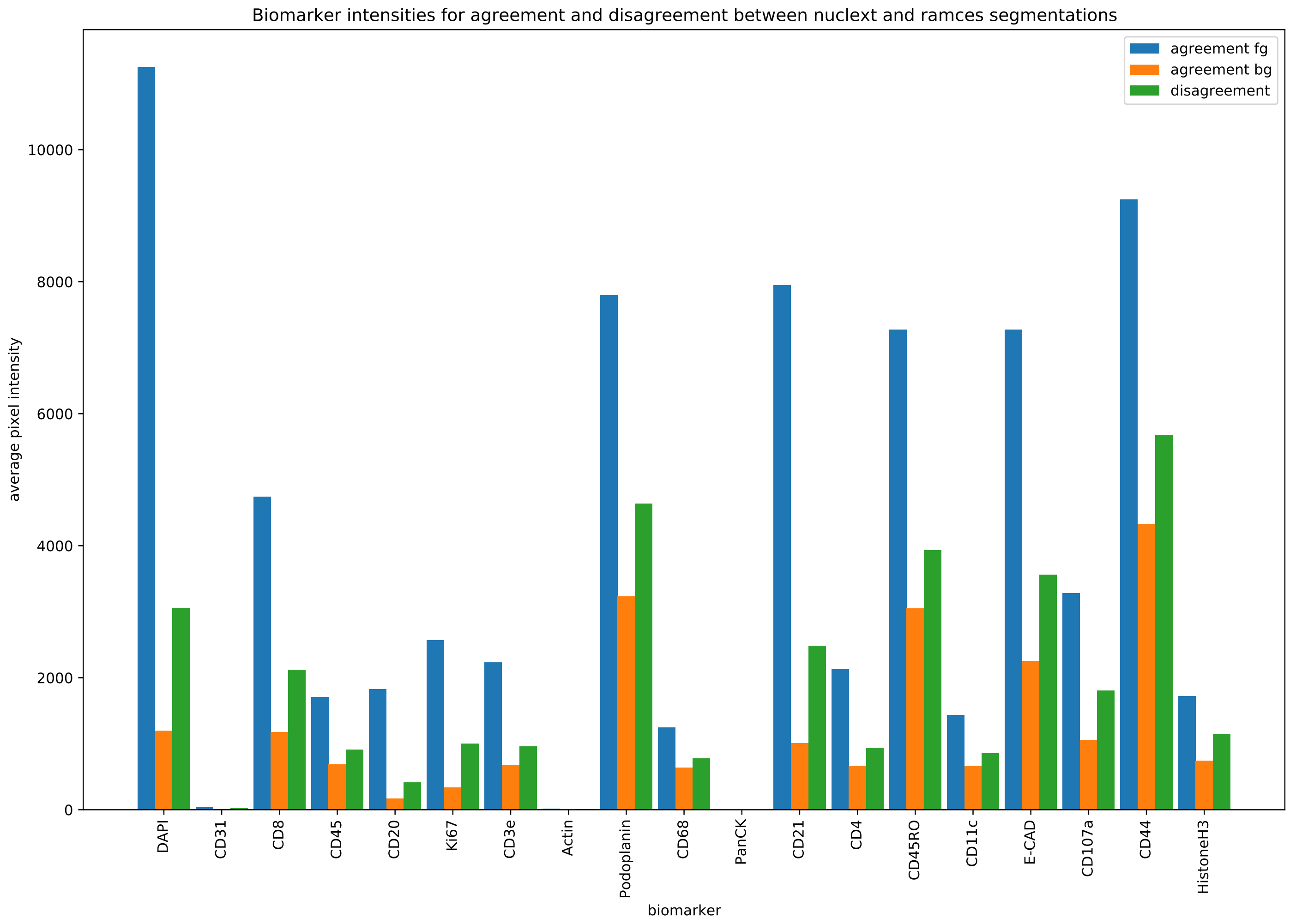

Supplementary Figure 5: Agreements and disagreements between the RAMCES segmentations (using the RAMCES combined output with the top 3 ranked markers) and the default nucleus extension segmentations for dataset 5 (Supplementary Table 1). The blue bars show the average pixel intensity in the areas where the two segmentation methods agree in the foreground (fg, inside of the cells). The orange bars show the segmentation agreement between the two segmentation methods in the background (bg, outside of the cells). The green bars show the segmentation disagreement.

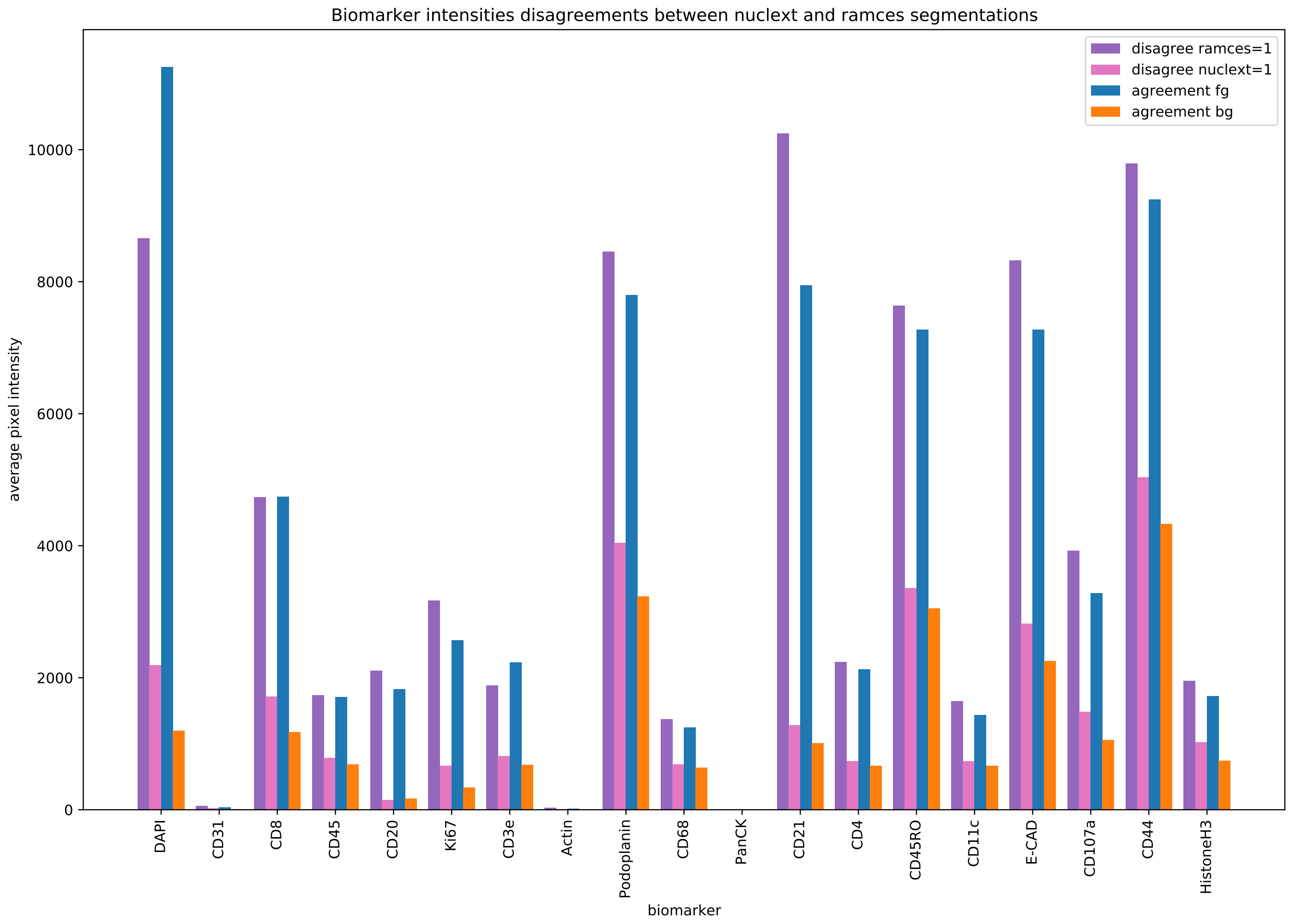

Supplementary Figure 6: Agreements and disagreements between the RAMCES segmentations (using the RAMCES combined output with the top 3 ranked markers) and the default nucleus extension segmentations for dataset 5 (Supplementary Table 1). The purple bars show average pixel intensity of biomarkers where RAMCES segmentations label a cell and the nucleus extension segmentations do not (‘disagree ramces=1’), and the pink bars show where the nucleus extension segmentations label a cell and RAMCES segmentations do not (‘disagree nuclext=1’). The blue and orange bars are the same as in Supplementary Figure 5.

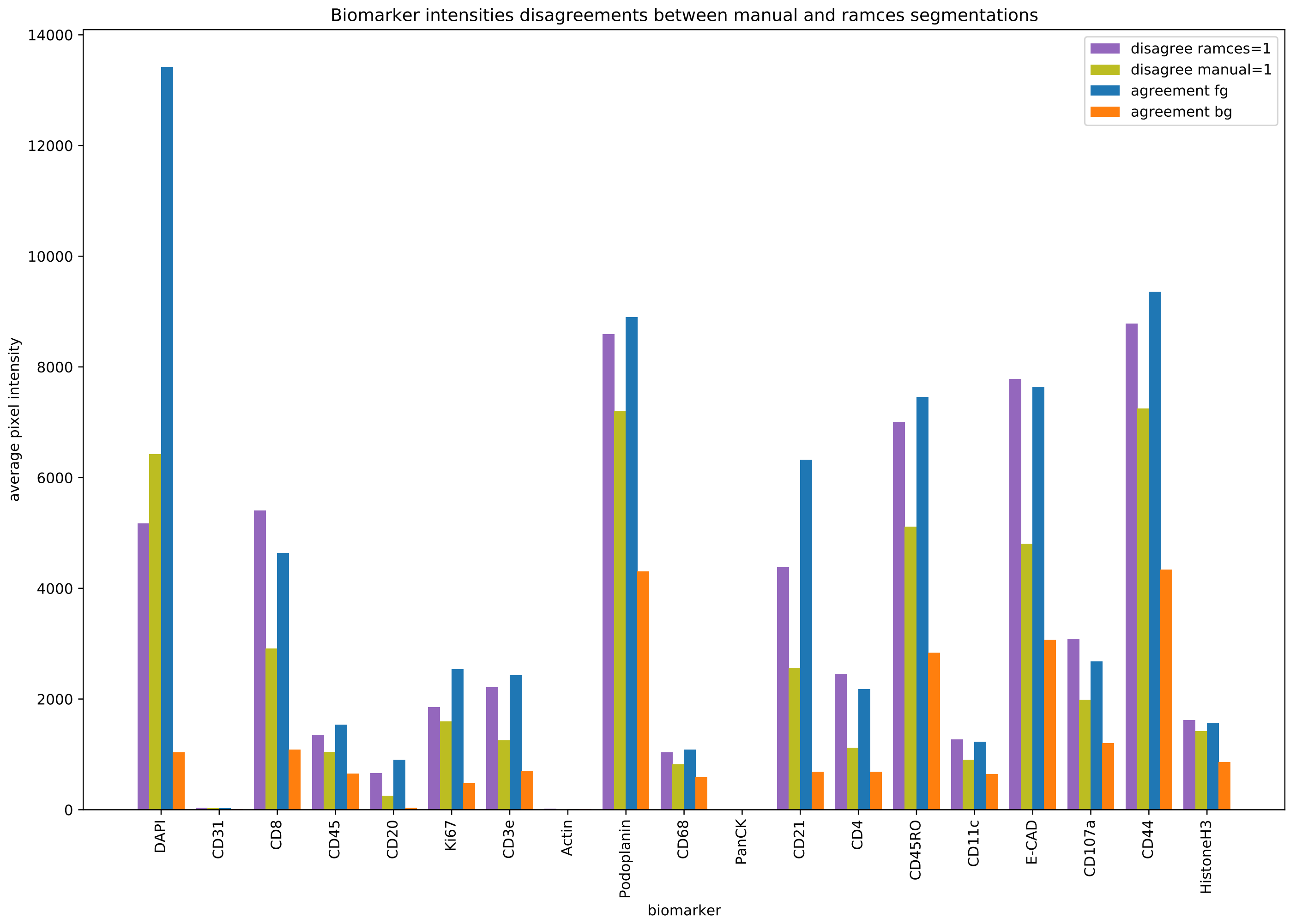

Supplementary Figure 7: Agreements and disagreements between the RAMCES segmentations (using the RAMCES combined output with the top 3 ranked markers) and the expert #1 annotations for the two manually annotated tiles from dataset 5 (Supplementary Table 1). The purple bars show average pixel intensity of biomarkers where RAMCES segmentations label a cell and the manual segmentations do not ('disagree ramces=1'), and the yellow bars show where the manual segmentations label a cell and RAMCES segmentations do not ('disagree manual=1'). The blue bars show the average biomarker intensities in areas where the two segmentation methods agree inside of cells, and the orange bars show average intensities where they agree in the background.

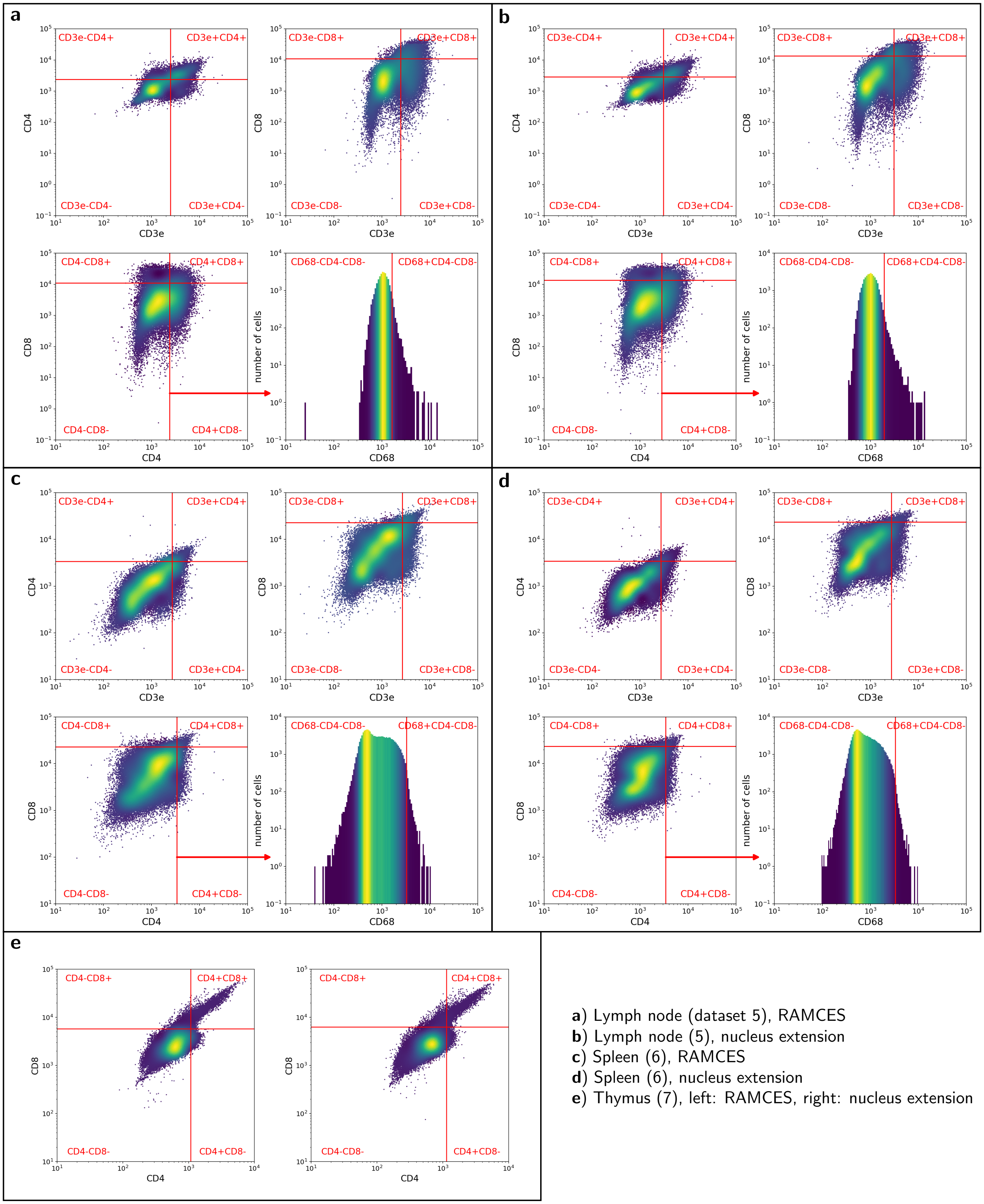

a) Lymph node (dataset 5), RAMCES
b) Lymph node (5), nucleus extension
c) Spleen (6), RAMCES
d) Spleen (6), nucleus extension
e) Thymus (7), left: RAMCES, right: nucleus extension

Supplementary Figure 8: Gating strategy for the percentage values in Supplementary Table 5. Thresholds are calculated using the $\mu + 2\sigma$ of the background intensity values for each specified channel (Methods). The legend on the bottom right indicates which dataset (Supplementary Table 1) and segmentation type each of the plots correspond to.

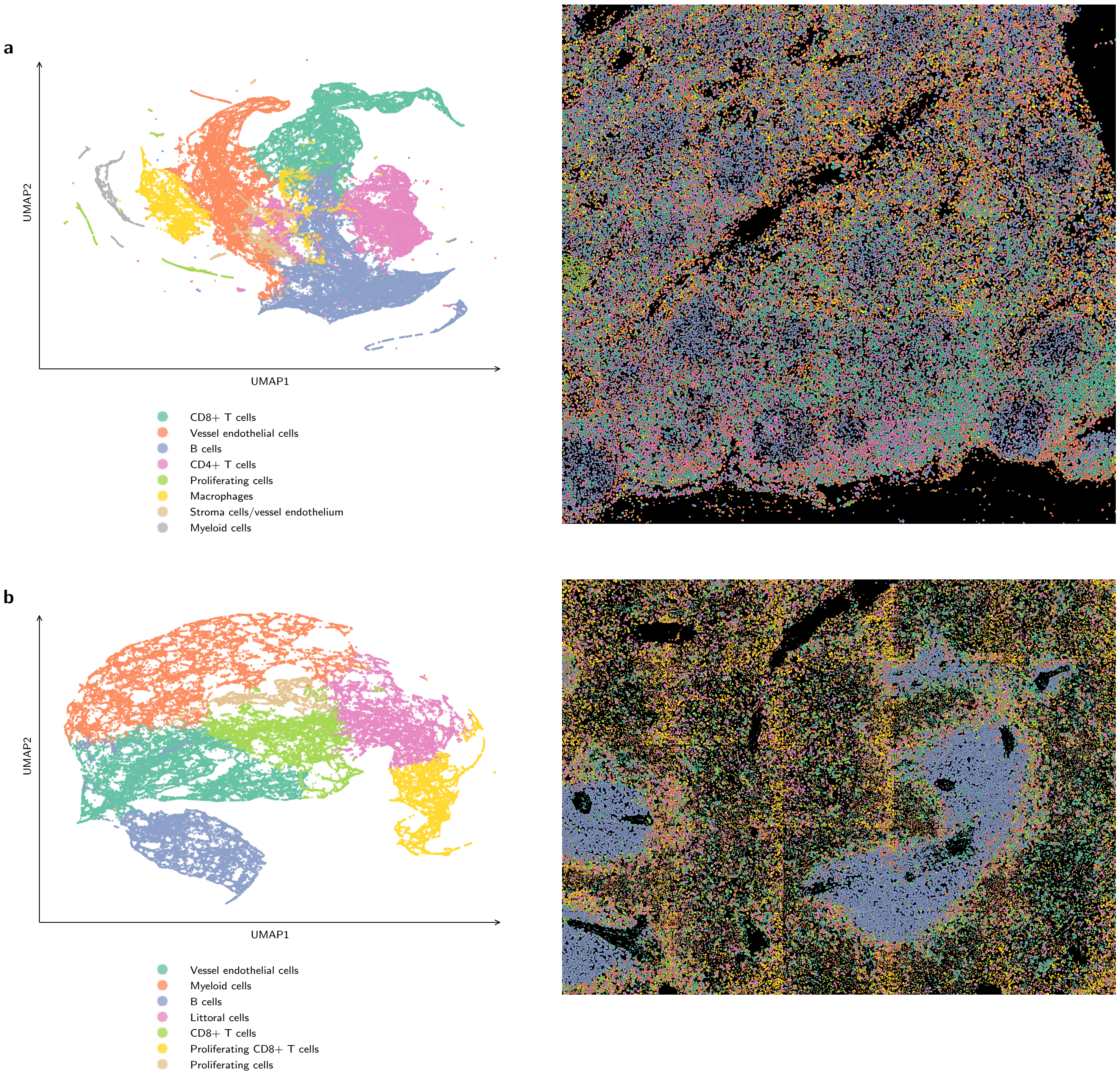

Supplementary Figure 9: Spatial assignment of cell types. UMAP embeddings and color legend indicating cell types and stitched tiles containing segmented cells from a) lymph node dataset 8 and b) spleen dataset 9 (Supplementary Table 1).

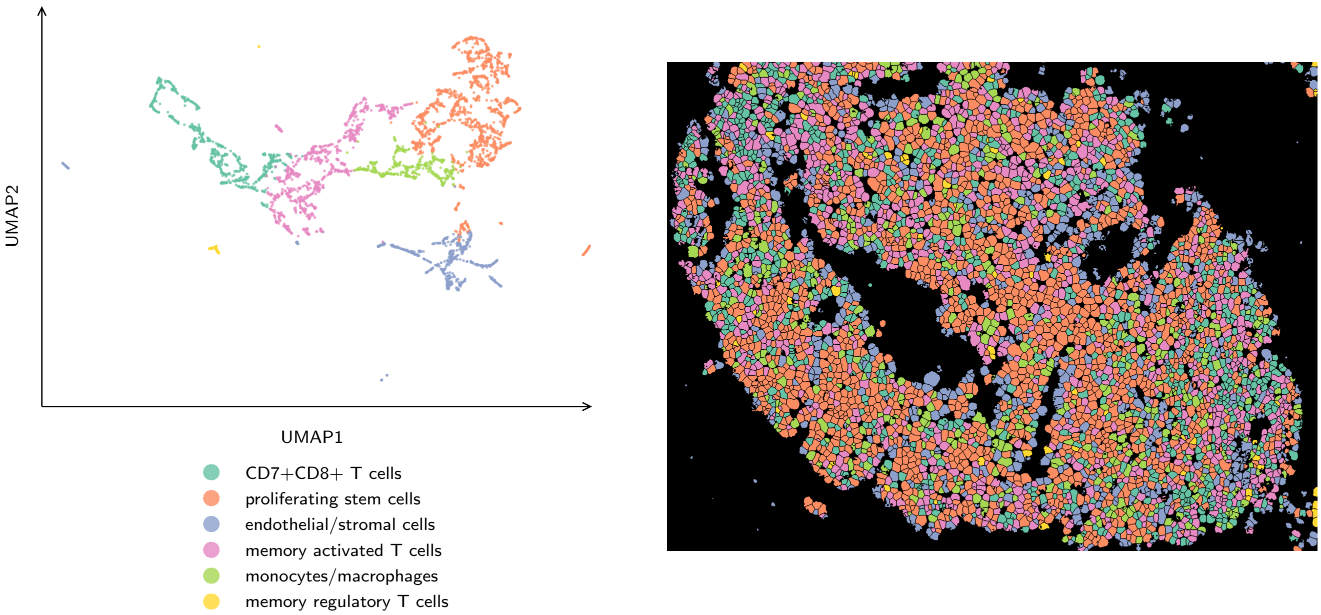

Supplementary Figure 10: Spatial assignment of cell types. UMAP embeddings and color legend indicating cell types and stitched tiles containing segmented cells from cancerous bone marrow dataset 11 (Supplementary Table 1).

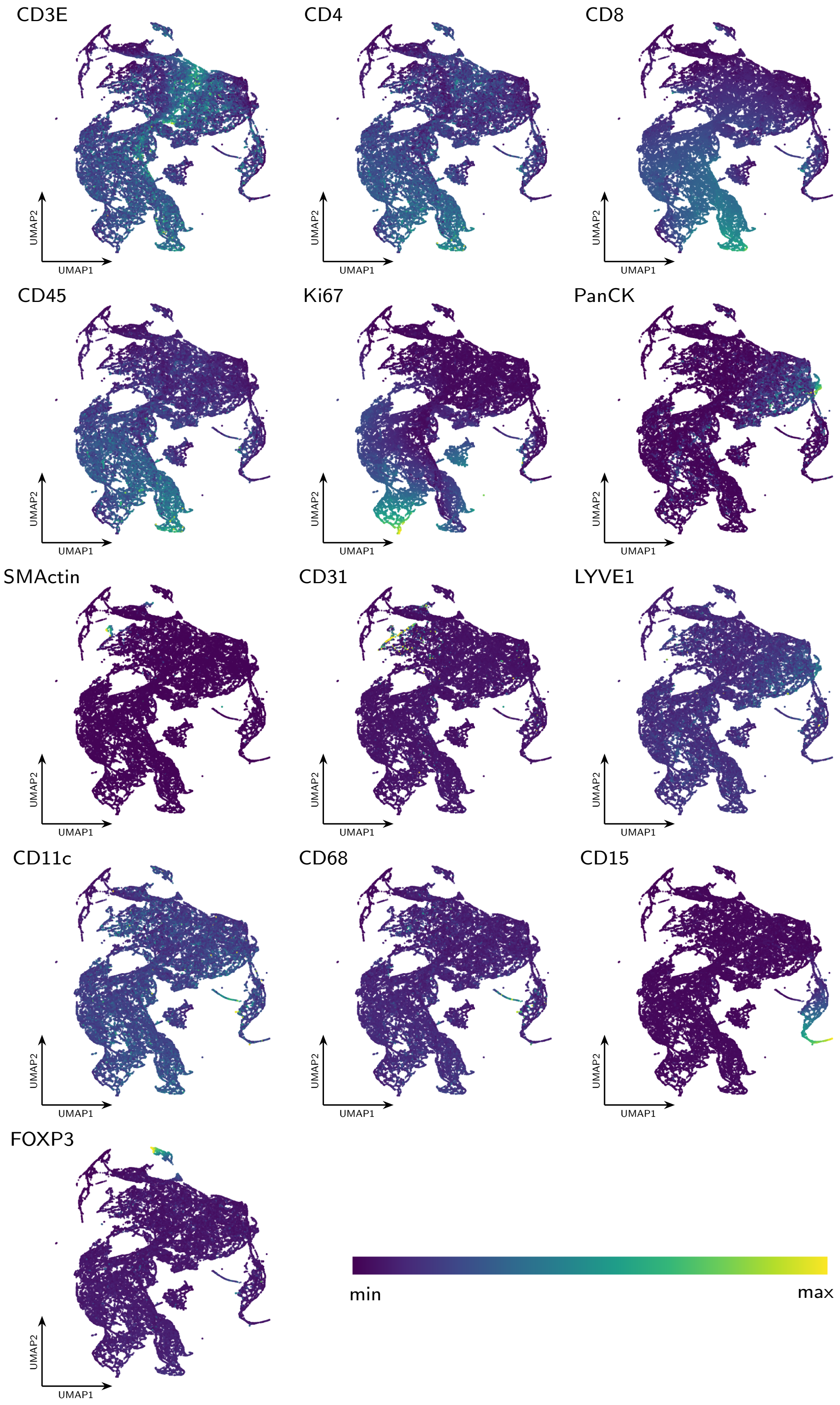

Supplementary Figure 11: UMAP cell embeddings showing key protein markers in the thymus dataset 10 (Supplementary Table 1), colored by marker abundance. Plots produced with the Cellar tool [5].

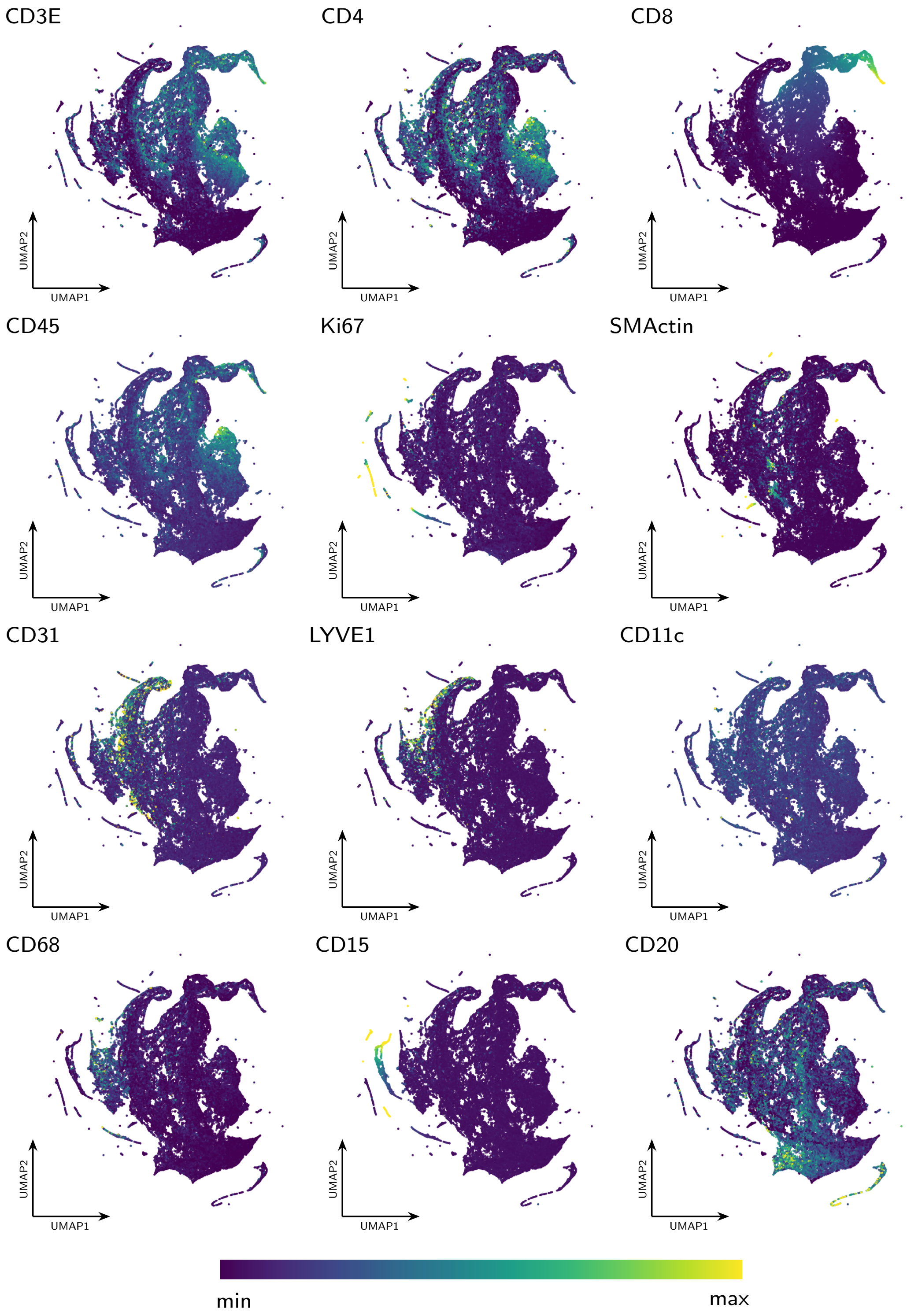

Supplementary Figure 12: UMAP cell embeddings showing key protein markers in the lymph node dataset 8 (Supplementary Table 1), colored by marker abundance. Plots produced with the Cellar tool [5].

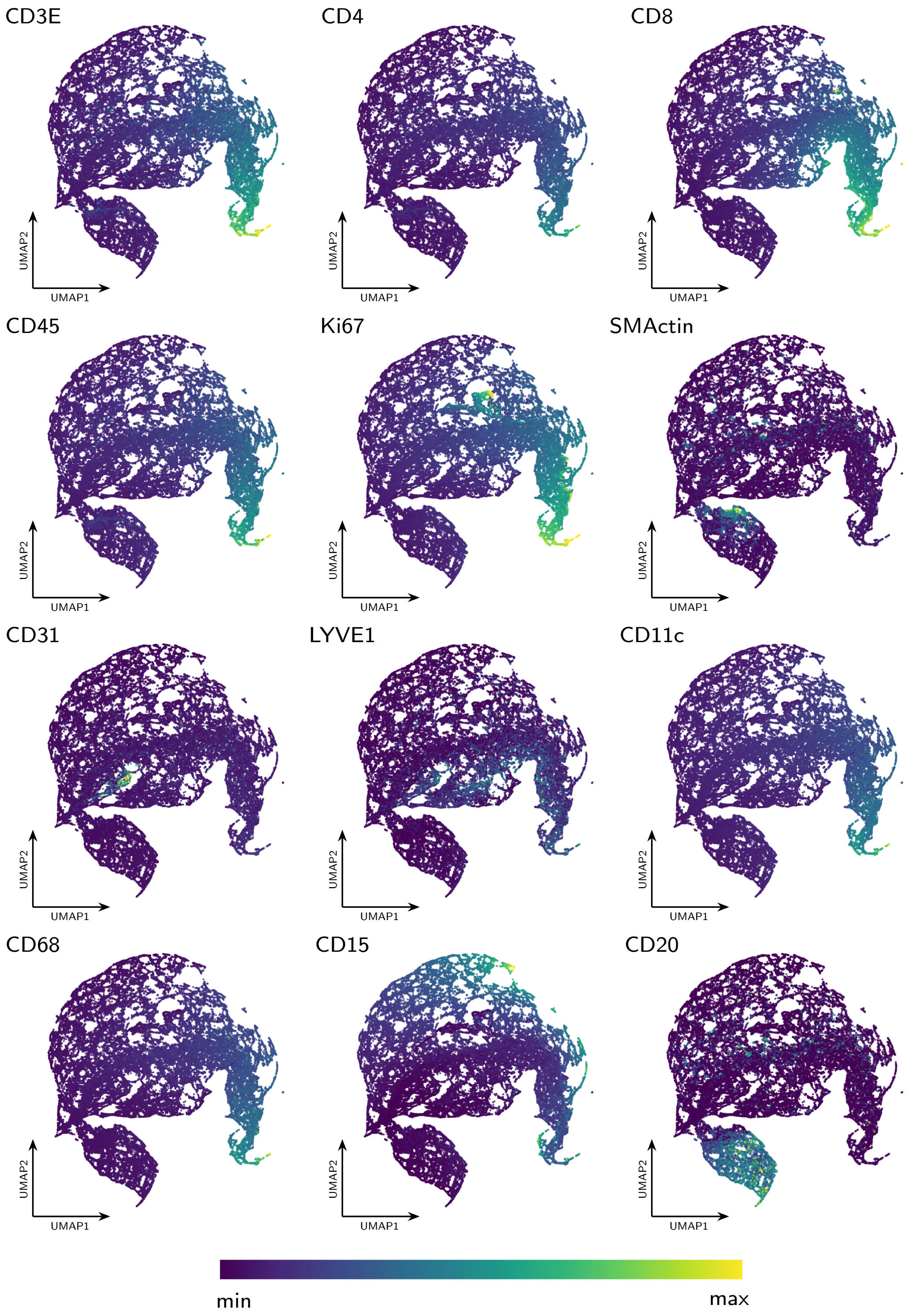

Supplementary Figure 13: UMAP cell embeddings showing key protein markers in the spleen dataset 9 (Supplementary Table 1), colored by marker abundance. Plots produced with the Cellar tool [5].

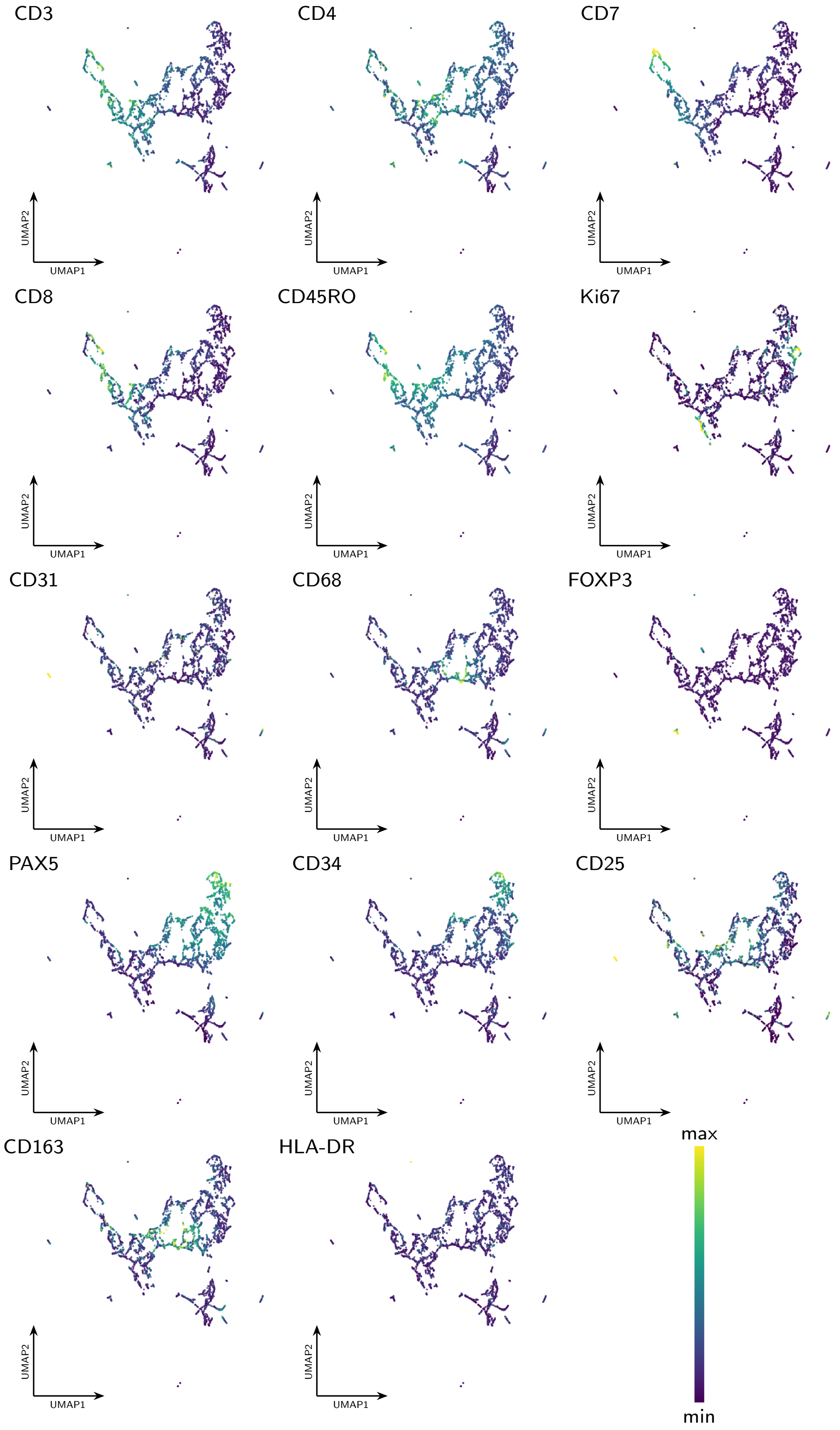

Supplementary Figure 14: UMAP cell embeddings showing key protein markers in the bone marrow dataset 11 (Supplementary Table 1), colored by marker abundance. Plots produced with the Cellar tool [5].

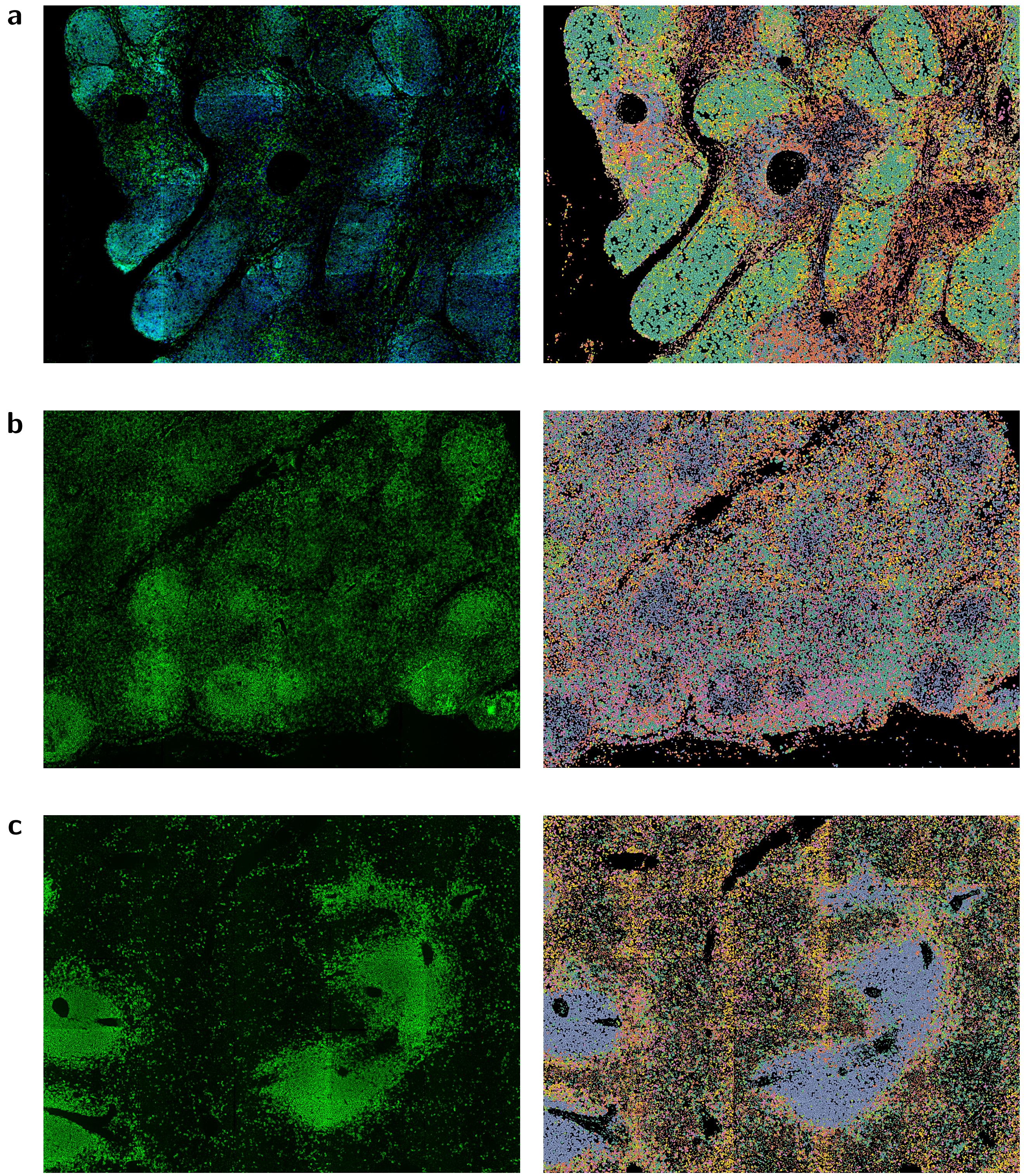

Supplementary Figure 15: CODEX images colored by select protein channels (left) with corresponding segmentation tiles colored by assigned cell type (right). a) Thymus dataset 10 showing CD4 and CD8 (green and blue, respectively, with cyan as the overlap, left), indicating presence of CD4+CD8+ T cells (green, right). b) Lymph node dataset 8 showing CD20 (green,left), indicating presence of CD20+ B cells (blue, right). c) Spleen dataset 9 showing CD20 (green, left), indicating presence of CD20+ B cells (blue, right).

channel_id	antibody_name	rr_id	uniprot_accession _number	lot_number	dilution	conjugated_cat _number	conjugated_tag	vendor
Cycle2_CH2	Anti-CD31 antibody	AB_1267039	P16284	B310793	1/200	Akoya 4450017	Akoya BX001-Alexa Fluor 750	Akoya
Cycle2_CH3	Anti-CD8a antibody	AB_2650657	P01732	B304054	1/200	Akoya 4250012	Akoya BX026-Atto 550	Akoya
Cycle3_CH2	Anti-CD20 antibody	AB_10734340	P11836	B310936	1/200	Akoya 4450018	Akoya BX007-Alexa Fluor 750	Akoya
Cycle3_CH3	Anti-Ki67 antibody	AB_396287	P46013	B305585	1/200	Akoya 4250019	Akoya BX047-Atto 550	Akoya
Cycle3_CH4	Anti-CD3e antibody	AB_764498	P07766	B320435	1/200	Akoya 4450030	Akoya BX045-Cy5	Akoya
Cycle4_CH2	Anti-SMA antibody	AB_2223019	P62736	UF-31Jul2020	1/200	ab240654	Akoya BX013-Alexa Fluor 750	Abcam
Cycle4_CH3	Anti-Podoplanin antibody	AB_1595616	Q86YL7	B319036	1/200	Akoya 4250004	Akoya BX023-Atto 550	Akoya
Cycle4_CH4	Anti-CD68 antibody	AB_11151139	P34810	B319545	1/200	Akoya 4350019	Akoya BX015-Cy5	Akoya
Cycle5_CH2	Anti-PanCK antibody	AB_2616960	P04264	B304089	1/200	Akoya 4450020	Akoya BX019-Alexa Fluor 750	Akoya
Cycle5_CH3	Anti-CD21 antibody	AB_1267035	P20023	UF-21Oct2020	1/200	ab193554	Akoya BX032-Atto 550	Abcam
Cycle5_CH4	Anti-CD4 antibody	AB_2750883	P01730	B320436	1/200	Akoya 4350018	Akoya BX004-Cy5	Akoya
Cycle6_CH2	Anti-Lyve1 antibody	AB_2884014	Q9Y5Y7	UF-12Feb2020	1/50	ab232935	Akoya BX004-Alexa Fluor 750	Abcam
Cycle6_CH3	Anti-CD45RO antibody	AB_314418	P08575	B311690	1/200	Akoya 4250023	Akoya BX017-Atto 550	Akoya
Cycle6_CH4	Anti-CD11c antibody	AB_2572997	P20702	B304058	1/200	Akoya 4350020	Akoya BX024-Cy5	Akoya
Cycle7_CH2	Anti-CD35 antibody	AB_2884017	P17927	UF-14Oct2020	1/100	ab240961	Akoya BX016-Alexa Fluor 750	Abcam
Cycle7_CH3	Anti-eCAD antibody	AB_2533118	P12830	B317026	1/200	Akoya 4250021	Akoya BX014-Atto 550	Akoya
Cycle7_CH4	Anti-CD107a antibody	AB_1134260	P11279	B319036	1/200	Akoya 4350001	Akoya BX006-Cy5	Akoya
Cycle8_CH2	Anti-CD34 antibody	AB_2861355	P28906	UF-14Oct2020	1/100	NBP2-32932	Akoya BX022-Alexa Fluor 750	Novus Bio
Cycle8_CH3	Anti-CD44 antibody	AB_312952	P16070	B315320	1/200	Akoya 4250002	Akoya BX005-Atto 550	Akoya
Cycle8_CH4	Anti-HLADR antibody	AB_10563656	P04233	B292265	1/200	Akoya 4450029	Akoya BX033-Cy5	Akoya
Cycle9_CH3	Anti-Foxp3 antibody	AB_467556	Q9BZS1	UF-12Feb2020	1/50	14-4777-82	Akoya BX020-Atto 550	eBioscience
Cycle9_CH4	Anti-CD163 antibody	AB_714951	Q86VB7	UF-03Aug2020	1/200	NBI10-40686	Akoya BX036-Cy5	Novus Bio
Cycle10_CH3	Anti-COL4 antibody	AB_2801511	P02462	UF-03Aug2020	1/200	ab226485	Akoya BX029-Atto 550	Abcam
Cycle10_CH4	Anti-Vimentin antibody	AB_306907	P08670	UF-31Jul2020	1/400	ab8978	Akoya BX042-Cy5	Abcam
Cycle11_CH3	Anti-CD15 antibody	AB_397181	P22083	UF-14Oct2020	1/200	559045	Akoya BX035-Atto 550	BD Biosciences
Cycle11_CH4	Anti-CD45 antibody	AB_11063696	P08575	UF-13Oct2020	1/100	14-9457-82	Akoya BX027-Cy5	eBioscience
Cycle12_CH3	Anti-CD5 antibody	AB_2884016	P06127	UF-13Oct2020	1/200	ab213003	Akoya BX041-Atto 550	Abcam
Cycle12_CH4	Anti-CD1c antibody	AB_2884015	P29017	UF-21Oct2020	1/100	ab270797	Akoya BX-30-Cy5	Abcam

Supplementary Table 10: Antibody information for University of Florida CODEX datasets. channel_id: structure of channel_id depends on assay type. antibody_name: anti-(target name) antibody. Not validated or used down-stream. rr_id: a unique antibody identifier that comes from the Antibody Registry (https://antibodyregistry.org). uniprot_accession_number: a unique identifier for proteins in the UniProt database (https://www.uniprot.org). lot_number: specific to the vendor. (e.g. Abcam lot# GR3238979-1). dilution: antibody solutions may be diluted according to the experimental protocol. conjugated_cat_number: an antibody may be conjugated to a fluorescent tag or metal tag for detection. Conjugated antibodies may be purchased from commercial providers. conjugated_tag: the name of the entity conjugated to the antibody.

Supplementary References

[1] Goltsev, Y. *et al.* Deep profiling of mouse splenic architecture with codex multiplexed imaging. *Cell* **174**, 968–981 (2018).

[2] Schürch, C. M. *et al.* Coordinated cellular neighborhoods orchestrate antitumoral immunity at the colorectal cancer invasive front. *Cell* **182**, 1341–1359 (2020).

[3] Uhlén, M. *et al.* Tissue-based map of the human proteome. *Science* **347** (2015).

[4] Czech, E., Aksoy, B. A., Aksoy, P. & Hammerbacher, J. Cytokit: A single-cell analysis toolkit for high dimensional fluorescent microscopy imaging. *BMC bioinformatics* **20**, 1–13 (2019).

[5] Hasanaj, E., Wang, J., Sarathi, A., Ding, J. & Bar-Joseph, Z. Cellar: Interactive single cell data annotation tool. Preprint at https://doi.org/10.1101/2021.03.19.436162 (2021).
