# Supplementary material for: Membrane marker selection for segmenting single cell spatial proteomics data
Source: Nat Commun. 2022 Apr 14;13:1999. doi: 10.1038/s41467-022-29667-w (PMC9010440; doi:10.1038/s41467-022-29667-w)
Supplement: Supplementary file 3 — Reporting Summary [file 41467_2022_29667_MOESM3_ESM.pdf]

nature portfolio

Corresponding author(s): Ziv Bar-Joseph

Last updated by author(s): Mar 15, 2022

Reporting Summary

Nature Portfolio wishes to improve the reproducibility of the work that we publish. This form provides structure for consistency and transparency in reporting. For further information on Nature Portfolio policies, see our Editorial Policies and the Editorial Policy Checklist.

Statistics

For all statistical analyses, confirm that the following items are present in the figure legend, table legend, main text, or Methods section.

n/a	Confirmed	
☐	☒	The exact sample size (n) for each experimental group/condition, given as a discrete number and unit of measurement
☒	☐	A statement on whether measurements were taken from distinct samples or whether the same sample was measured repeatedly
☒	☐	The statistical test(s) used AND whether they are one- or two-sided<br>*Only common tests should be described solely by name; describe more complex techniques in the Methods section.*
☒	☐	A description of all covariates tested
☒	☐	A description of any assumptions or corrections, such as tests of normality and adjustment for multiple comparisons
☐	☒	A full description of the statistical parameters including central tendency (e.g. means) or other basic estimates (e.g. regression coefficient) AND variation (e.g. standard deviation) or associated estimates of uncertainty (e.g. confidence intervals)
☒	☐	For null hypothesis testing, the test statistic (e.g. F, t, r) with confidence intervals, effect sizes, degrees of freedom and P value noted<br>*Give P values as exact values whenever suitable.*
☒	☐	For Bayesian analysis, information on the choice of priors and Markov chain Monte Carlo settings
☒	☐	For hierarchical and complex designs, identification of the appropriate level for tests and full reporting of outcomes
☒	☐	Estimates of effect sizes (e.g. Cohen's d, Pearson's r), indicating how they were calculated

Our web collection on statistics for biologists contains articles on many of the points above.

Software and code

Policy information about availability of computer code

Data collection	CODEX (now PhenoCycler) software suite, version 1.30.0.12. Akoya BIOSCIENCES https://www.akoyabio.com/phenocycler/software/
Data analysis	Code for data analysis can be found at https://github.com/mdayao/ramces/<br>PyTorch (>= v1.0.0) was used to train and evaluate the RAMCES CNN model. The Weights & Biases package (CLI v0.14.2) was used to visualize training metrics. The pywavelets package (>=v1.1.1) was used for performing the DWT during pre-processing. The Cytokit software (version 'latest' from https://hub.docker.com/r/eczech/cytokit, uploaded on Feb 5, 2020) was used for raw image processing and cell segmentation.

For manuscripts utilizing custom algorithms or software that are central to the research but not yet described in published literature, software must be made available to editors and reviewers. We strongly encourage code deposition in a community repository (e.g. GitHub). See the Nature Portfolio guidelines for submitting code & software for further information.

Data

Policy information about availability of data

All manuscripts must include a data availability statement. This statement should provide the following information, where applicable:

- Accession codes, unique identifiers, or web links for publicly available datasets
- A description of any restrictions on data availability
- For clinical datasets or third party data, please ensure that the statement adheres to our policy

The HuBMAP data used in this study are available in the HuBMAP data portal [https://portal.hubmapconsortium.org] with HuBMAP IDs HBM869.VZJM.366 [https://portal.hubmapconsortium.org/browse/dataset/a6ccc344f88a164766d1251053173009], HBM432.LLCF.677 [https://portal.hubmapconsortium.org/browse/dataset/75edcda4f3ff5bef72383d5d082438c2], HBM588.FHDS.363 [https://portal.hubmapconsortium.org/browse/dataset/8dd0ef5cafa3541cf9f0661db64662b7],

HBM279.TQRS.775 [https://portal.hubmapconsortium.org/browse/dataset/077f7862f6306055899374c7807a30c3], HBM337.FSXL.564 [https://portal.hubmapconsortium.org/browse/dataset/f0c58e670ceb445e6ab02c6a20c83aee], HBM376.QCCJ.269 [https://portal.hubmapconsortium.org/browse/dataset/4514230f7473a496201a4e45c4ff9568], HBM754.WKLP.262 [https://portal.hubmapconsortium.org/browse/dataset/c95d9373d698faf60a66ffdc27499fe1], HBM556.KSFB.592 [https://portal.hubmapconsortium.org/browse/dataset/00d1a3623dac388773bc7780fcb42797], HBM288.XSQZ.633 [https://portal.hubmapconsortium.org/browse/dataset/f86b9efc87074bf03cd53932d8f1e76f].
Segmentation masks and RAMCES outputs are available in Zenodo with the identifier doi:10.5281/zenodo.5655738.
Celltype annotations can be found at the Cellar tool [https://data.test.hubmapconsortium.org/app/cellar].
Processed primary imaging data for the bone marrow dataset was from Schurch et al. (2020) from the `Multi-tumor TMA' data, region 4. The primary imaging data for the mouse spleen dataset was from Goltsev et al. (2019).

Field-specific reporting

Please select the one below that is the best fit for your research. If you are not sure, read the appropriate sections before making your selection.

☒ Life sciences ☐ Behavioural & social sciences ☐ Ecological, evolutionary & environmental sciences

For a reference copy of the document with all sections, see nature.com/documents/nr-reporting-summary-flat.pdf

Life sciences study design

All studies must disclose on these points even when the disclosure is negative.

Sample size	We used 9 human tissue samples, for each we profiled the entire section. 3 of the datasets, one each from human lymph node, spleen and thymus, were used for training the RAMCES model. These datasets profiled 19 distinct markers 6 additional datasets (2 from each aforementioned tissue) were used for evaluation of the model and the resulting segmentations. Of those 6 datasets, 3 profiled 19 distinct markers and the other 3 profiled 29. These datasets were chosen to demonstrate RAMCES' ability to generalize to datasets with unseen protein markers. Further analysis was performed with 2 previously published datasets, one of the mouse spleen from Goltsev et al. (2019) and one of bone marrow from Schurch et al. (2020).
Data exclusions	No data was excluded.
Replication	For determining the ranking of markers for each dataset, RAMCES was run on at least 2 different processors, which verified that the outputs of RAMCES were consistent and without errors. For comparisons to manual segmentations, two tiles were manually segmented and compared to (Supplementary Tables 7-9, Supplementary Figure 7). Comparisons to both tiles led to the same conclusion: RAMCES-based segmentation outperformed nucleus extension segmentation as well as segmentation based on just 1 marker alone. For all other analysis in this work, replication is not relevant as the analysis is computational and deterministic.
Randomization	Not relevant. We looked at all cells in the dataset.
Blinding	Not relevant. Tissues were selected independent of the outcome.

Reporting for specific materials, systems and methods

We require information from authors about some types of materials, experimental systems and methods used in many studies. Here, indicate whether each material, system or method listed is relevant to your study. If you are not sure if a list item applies to your research, read the appropriate section before selecting a response.

Materials & experimental systems

n/a	Involved in the study	
☐	☒	Antibodies
☒	☐	Eukaryotic cell lines
☒	☐	Palaeontology and archaeology
☒	☐	Animals and other organisms
☐	☒	Human research participants
☒	☐	Clinical data
☒	☐	Dual use research of concern

Methods

n/a	Involved in the study	
☒	☐	ChIP-seq
☒	☐	Flow cytometry
☒	☐	MRI-based neuroimaging

Antibodies

Antibodies used	Information for all antibodies can be found in Supplementary Table 10.
Validation	https://www.akoyabio.com/phenocycler/assays/

Human research participants

Policy information about studies involving human research participants

Population characteristics	Donor demographic information is available on the HUBMAP portal (https://portal.hubmapconsortium.org/) through the dataset IDs provided in Supplementary Table 1. To summarize, this work included 7 donors: age mean 13.3, range 1-21 yrs; female ratio 28.6%; race ratio: 42.9% African American, 57.1% non-Hispanic White.
Recruitment	Organ donor tissue samples were recovered by the HuBMAP Lymphatic System Tissue Mapping Center (TMC) according to established protocols (dx.doi.org/10.17504/protocols.io.bsdsna6e) approved by the University of Florida Institutional Review Board (IRB201600029), the United Network for Organ Sharing (UNOS), in accordance with federal guidelines, and with written informed consent from each donor's legal representative. The studies were conducted in accordance with the relevant criteria set forth in the Declaration of Helsinki.
Ethics oversight	The University of Florida Institutional Review Board (IRB201600029) approved the study protocol.

Note that full information on the approval of the study protocol must also be provided in the manuscript.
